# Reliability-based load and resistance factor design model for energy piles

**DOI:** 10.1038/s41598-022-19142-3

**Published:** 2022-08-29

**Authors:** Biao Hu, Quanmei Gong, Yueqiang Zhang, Yihe Yin, Wenjun Chen

**Affiliations:** 1 College of Physics and Optoelectronic Engineering, Shenzhen University, Shenzhen, 518060 China; 2 Shanghai Key Laboratory of Rail Infrastructure Durability and System Safety, Tongji University, Shanghai, 201804 China; 3 The Key Laboratory of Road and Traffic Engineering, Ministry of Education, Tongji University, Shanghai, 201804 China

**Keywords:** Engineering, Civil engineering

## Abstract

Energy piles have been popular globally with functions of both pile foundation and ground source heat pumps. Although several researches have been devoted to the probabilistic design and assessment of energy piles, the corresponding procedures are too complicated for engineers. As a simple variant of the reliability-based design method, the load and resistance factor design (LRFD) approach for the geotechnical design of energy piles is presented in this study. Firstly, the load-transfer model for energy piles is developed to investigate the effect of cyclic thermal loading on the pile settlement. Then, the LRFD procedures based on first-order reliability method and target reliability method are implemented into two different constrained nonlinear optimization problems, respectively. The proposed LRFD model for energy piles is demonstrated through an example pile and a series of parametric analyses.

## Introduction

Geothermal energy, as one of the renewable energies, is rich in China. To improve the energy utilization structure and protect the environment, the utilization of the geothermal energy has been promoted heavily. Ground source heat pumps (GSHP) system have been proved as a mature technology to use the shallow geothermal energy. In recent years, energy piles have been popular globally with functions of both pile foundation and GSHP. In practice, energy piles are typically precast or cast-in-place piles. The heat-transfer pipes are placed in the pile and made of high-density polyethylene. The diameter and wall thickness of the pipe are 10–40 mm and 2–4 mm, respectively. In cool climates or cold seasons, the heat present in the ground is typically extracted and transferred to the superstructure, while the heat is typically extracted from the superstructure and injected into the ground in warm climates or during hot seasons^1^.

Several researches have suggested that the thermal loading has a significant effect on the response of energy piles (e.g.,^2–4^), especially for their long-term performance (e.g.,^5–7^). Thus, besides satisfying the design requirements for traditional piles, the thermal loading should be properly considered in the design of energy piles. Several analysis methods have been developed to investigate the thermomechanical behavior of energy piles, such as the empirical method (e.g.,^8–10^), load-transfer method (e.g.,^11–13^), and Full numerical method (e.g.,^14–16^). However, it is still difficult to ascertain the pile performance in the geotechnical design of energy piles due to the uncertainties in soil parameters, applied load, design model, etc. In the traditional deterministic design approach, a conservative factor of safety (FS) is typically used to avoid the effect of these uncertainties, where those uncertainties are not explicitly quantified. For example, to prevent any potential thermally-induced damage to the building, the FS value of the in-situ energy pile was designed as 13 in Switzerland^4,17^.

As a more rational design approach, the reliability-based design method was recently adopted in the geotechnical design of energy piles^18–22^. In the context of reliability-based design, the performance of energy piles was investigated probabilistically. Based on the probabilistic method, the reliability index or probability of failure is typically calculated and selected as the measure of the uncertainties. By selecting a target reliability index or acceptable failure probability, the least-cost design that satisfies the safety requirement is typically selected as the final design. However, it is difficult to characterize the accurate statistics of these uncertainties due to the limited available data, which could further lead to an under-design or over-design of energy pile. To address those problems, a better model based on robust design method was proposed for energy piles, where the design robustness was considered to find a final design that is less sensitive to the uncertainties^23^. As a simple variant of the reliability-based design method, the load and resistance factor design (LRFD) approach is more popular in geotechnical practice (e.g.,^24,25^), whereas no relevant research has been reported on the energy piles.

To this end, this paper aims to present a reliability-based LRFD method for the geotechnical design of energy piles. Firstly, based on the load-transfer method, a deterministic design model considering the long-term performance of energy piles is presented. Then, the LRFD procedures based on first-order reliability method and target reliability method are described, respectively, which are implemented as constrained nonlinear optimization problems in this study. By solving the optimization problems, the partial factors of each random variable can be evaluated. Focusing on the energy pile failure due to excessive settlement, the application of this LRFD model for energy piles is illustrated through an example pile. Furthermore, a series of parametric analyses are conducted to investigate the feasibility of the proposed LRFD approach.

## Deterministic design model for energy piles

### Load-transfer model

**Figure 1:**
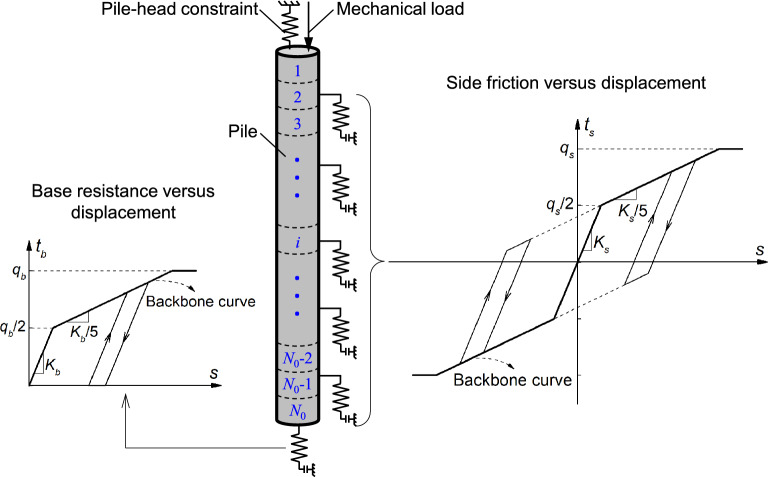
Illustration of load-transfer model for energy piles.

As a practical geotechnical design tool for traditional piles, the load-transfer method was firstly proposed by Coyle and Reese^26^ to investigate their stress and strain distributions along pile. Recently, the load-transfer method was further adopted in the geotechnical design of energy piles^12^. In the load-transfer analyses of energy piles, the tri-linear curve (e.g.,^12^) and hyperbolic curve (e.g.,^11^) are typically selected as the load-transfer curves. Taking the Frank and Zhao’s tri-linear curve^27^ as the backbone curve, Fig. 1 shows the load-transfer curves that represent the relationship between side friction (*t*_*s*_) [or base resistance (*t*_*b*_)] and pile-soil relative displacement (*s*). Note, the thermally-induced reloading and unloading paths of the load-transfer curves follow the Masing rules^28^. For fine-grained soils, the curve slopes can be determined as follows^27,29^:

1a $$K_{s} = \frac{{2E_{M} }}{D}$$

1b $$K_{b} = \frac{{11E_{M} }}{D}$$

where *K*_*s*_ and *K*_*b*_ are the slopes of the first linear branches for the shaft and base load-transfer curves (MPa/m) [as illustrated in Fig. 1], respectively; *E*_*M*_ is the Menard pressuremeter modulus (MPa), *D* is the pile diameter (m), and *q*_*s*_ and *q*_*b*_ are the ultimate side resistance and ultimate base resistance (MPa), respectively. It is noted that the values for *q*_*s*_ and *q*_*b*_ are typically calculated by the total stress method (*α*-method) or effective stress method (*β*-method) depending on the soil type.

In this study, the load-transfer model for energy piles is established using the matrix displacement method due to its good computational efficiency. As shown in Fig. 1, the pile is typically divided into *N*_0_ equal elements. Each element and node are numbered from top to bottom. Similar to the traditional piles, the static equilibrium equation of energy piles is expressed as follows:

2 $$\frac{dP\left( z \right)}{{dz}} = - T_{s} (z)$$

where *P*(*z*) is the axial force at the depth of *z* (kN), $T_{s} \left( z \right)$ is the side friction at the depth of *z* (kPa). Considering the effect of temperature change, the compression deformation of pile section (*ds*) is given as:

3 $$ds = \frac{P\left( z \right)dz}{{EA}} - \alpha \Delta Tdz$$

where *E* is the pile elastic modulus (GPa), *A* is the pile cross sectional area (m^2^), *α* is the thermal expansion/contraction coefficient of pile (°C^−1^), and Δ*T* is the temperature change (°C). Based on Eqs. (2) and (3), the local equilibrium of Element *i* (ranging from 1 to *N*_0_) can be obtained as:

4 $$\left[ {K_{P} } \right]_{i}^{e} \left\{ s \right\}_{i}^{e} = \left\{ P \right\}_{i}^{e} - \left\{ {T_{S} } \right\}_{i}^{e} - \left\{ {P_{T} } \right\}_{i}^{e}$$

5 $$\left[ {K_{P} } \right]_{i}^{e} = \left[ {\begin{array}{*{20}c} {\frac{EA}{{l_{i} }}} & { - \frac{EA}{{l_{i} }}} \\ { - \frac{EA}{{l_{i} }}} & {\frac{EA}{{l_{i} }}} \\ \end{array} } \right]$$

where $\left[ {K_{P} } \right]_{i}^{e}$ is the stiffness matrix of Element *i*, $\left\{ s \right\}_{i}^{e} = \left\{ {s_{i} ,\;s_{i + 1} } \right\}^{T}$ is the column vector of element node displacement, $\left\{ P \right\}_{i}^{e} = \left\{ {P_{i} ,\;P_{i + 1} } \right\}^{T}$ is the column vector of the external loads acting on element nodes, $\left\{ {T_{S} } \right\}_{i}^{e}$ is the column vector of the equivalent side resistance and/or tip resistance at nodes, $\left\{ {P_{T} } \right\}_{i}^{e} = \left\{ {EA\alpha \Delta T,\; - EA\alpha \Delta T} \right\}^{T}$ is the column vector of the thermally induced forces acting on element nodes, and *l*_*i*_ is the length of Element *i*. Considering the constraint effect of pile head and pile base, $\left\{ {T_{S} } \right\}_{i}^{e}$ is defined as follows:

6 $$\left\{ {T_{S} } \right\}_{i}^{e} = \left[ {K_{S} } \right]_{i}^{e} \left\{ s \right\}_{i}^{e} = \left[ {\begin{array}{*{20}c} {\frac{{Cl_{i} k_{s,i} }}{4} + \delta_{1i} AK_{h} } & {\frac{{Cl_{i} k_{s,i} }}{4}} \\ {\frac{{Cl_{i} k_{s,i} }}{4}} & {\frac{{Cl_{i} k_{s,i} }}{4} + \delta_{{N_{0} i}} Ak_{b} } \\ \end{array} } \right]\left\{ s \right\}_{i}^{e}$$

where $\left\{ {K_{S} } \right\}_{i}^{e}$ is the stiffness matrix on the pile-soil interface of Element *i*, *C* is the pile’s circumference (m), *k*_*s,i*_ and *k*_*b*_ are the secant slopes on the side and base load-transfer curves for a certain displacement (MPa/m), respectively, *K*_*h*_ is the pile-superstructure interaction stiffness (MPa/m), and *δ*_*ij*_ is the Kronecker delta (i.e., if *i* = *j*, *δ*_*ij*_ = 1; otherwise *δ*_*ij*_ = 0). Next, based on Eqs. (4)‒(6), the external equilibrium is expressed as:

7 $$\left( {\left[ {K_{P} } \right]{ + }\left[ {K_{S} } \right]} \right)\left\{ s \right\} = \left( {\left\{ P \right\} - \left\{ {P_{T} } \right\}} \right)$$

where $\left[ {K_{P} } \right]$ is the total stiffness matrix of pile, $\left[ {K_{S} } \right]$ is the total stiffness matrix on the pile-soil interface, $\left\{ s \right\} = \left\{ {s_{1} ,\;s_{2} , \ldots ,s_{{N_{0} }} ,\;s_{{N_{0} + 1}} } \right\}^{T}$ is the column vector of pile node displacement, $\left\{ P \right\} = \left\{ {P_{M} ,\;0, \ldots ,0,\;0} \right\}^{T}$ is the column vector of external loads, and $\left\{ {P_{T} } \right\} = \left\{ {EA\alpha \Delta T,\;0, \ldots ,0,\; - EA\alpha \Delta T} \right\}^{T}$ is the column vector of thermal loads. Equation (7) can be solved using the incremental iteration method. It is noted the pile response at the end of previous loading is selected as the initial state in the calculation of next loading. For more details, readers can refer to some recent publications such as Fei et al.^11^, Luo and Hu^19,23^ and Hu and Luo^18^.

### Serviceability limit state design

**Figure 2:**
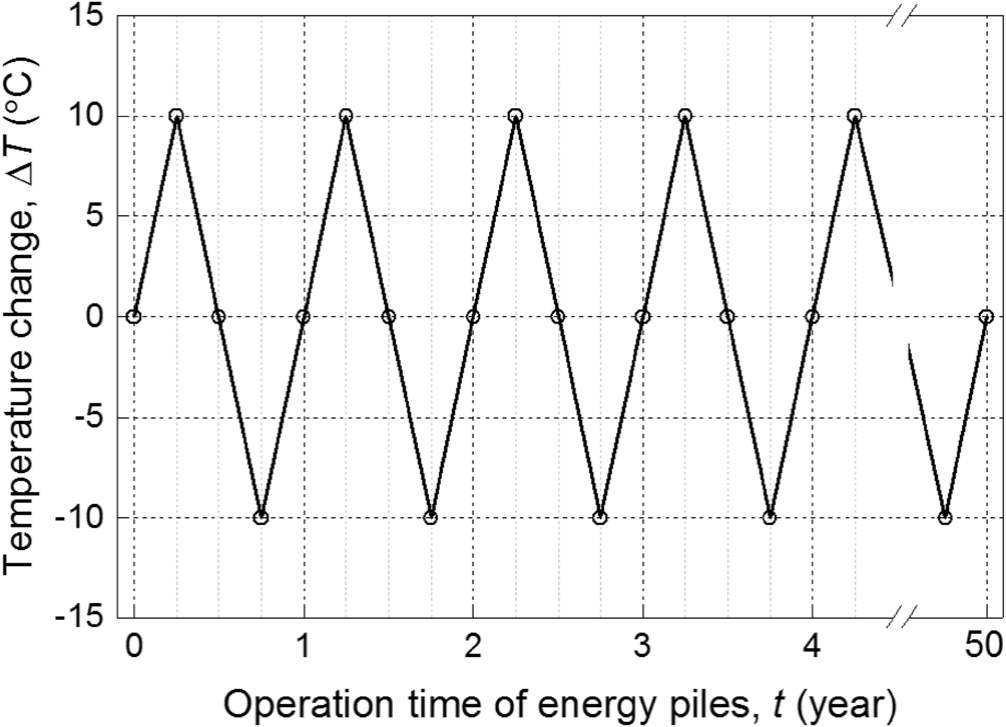
Temperature cycle schema of energy pile alternating summer and winter operation.

Several researches have been conducted to investigate the role of thermal loading in the geotechnical design of energy piles. It was suggested that the effect of thermal loading must be considered in the analysis and design of energy piles^10^. Compared with the ultimate limit state, the performance degradation of the energy piles due to the piping need in the serviceability limit state was suggested to be more considered in the design (e.g.,^6^). This paper focuses on the excessive settlement failure due to cyclic thermal loading. The temperature cycle mode has been suggested in our previous study^18^. Taking the energy pile alternating summer and winter operation as an example, Fig. 2 illustrates a typical temperature cycle with a maximum temperature change of ± 10 °C. Thus, the limit state function of excessive settlement can be defined:

8 $$g\left( x \right) = s_{\lim } - s_{1}$$

where *s*_lim_ is the limiting pile settlement (m), which is typically selected as 5% of pile diameter, *s*_1_ is the pile settlement that can be computed with the developed load-transfer model (m).

## Load and resistance factor design approach

Several methods are typically used for the back-calculation of partial factors in the load and resistance factor design (LRFD), including but not limited to first-order second-moment method (FOSM), Monte Carlo simulations (MCS), first-order reliability method (FORM) and target reliability method (TRM). Due to the merits of information on design point and parametric sensitivities^30^, both FORM and TRM are adopted herein to estimate the partial factors.

### First-order reliability method

Low and Tang^31^ presented spreadsheet-based practical procedures for FORM-based analysis and design. The reliability index (*β*) was defined as follows:

9 $$\beta = \mathop {\min }\limits_{{{\mathbf{x}} \in f}} \sqrt {{\mathbf{n}}^{T} {\mathbf{R}}^{ - 1} {\mathbf{n}}}$$

10 $$n_{i} = \frac{{x_{i} - \mu_{i}^{N} }}{{\sigma_{i}^{N} }} = \Phi^{ - 1} \left[ {F\left( {x_{i} } \right)} \right]$$

where **x** is the vector of random variables (*x*_*i*_), *f* is the failure domain, **n** is the unrotated equivalent standard normal vector of *n*_*i*_, **R** is the correlation matrix of the random variables, $\mu_{i}^{N}$ and $\sigma_{i}^{N}$ are the equivalent normal mean value and standard deviation of *x*_*i*_, respectively, Φ[] is the cumulative distribution function (CDF) of a standard normal distribution, *F*(*x*_*i*_) is the original nonnormal CDF evaluated at *x*_*i*_. As noted by Low and Tang^31^, *β* calculated by varying **n** is more efficient and robust than that determined by varying **x**. Hence, the **n**-space procedure^31^ is adopted in this study.

**Figure 3:**
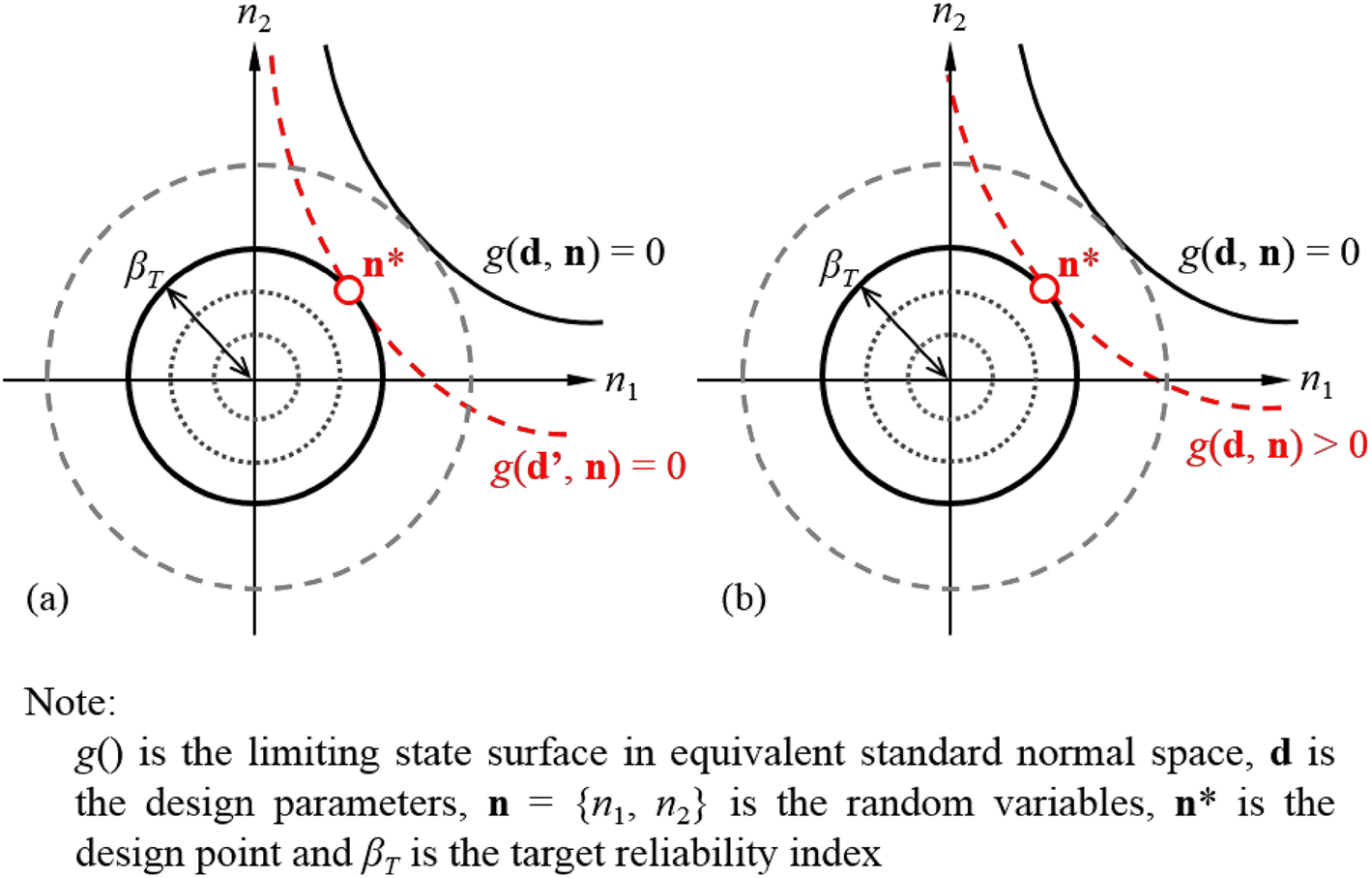
Determination of design point for target reliability index: (**a**) First-order reliability method; (**b**) Target reliability method.

Figure 3a illustrates the FORM-based procedure to determine the design point (n*) for target reliability index (*β*_*T*_). For a certain design scenario, the calculation of *β* is implemented as a constrained optimization problem^30^:

11 $$\begin{gathered} {\text{Find:}}\;\;{\mathbf{d}} \hfill \\ \;\;\;\;\;\;{\text{Find:}}\;\;{\mathbf{n}} \hfill \\ \;\;\;\;\;\;{\text{Subject}}\;{\text{to:}}\;\;g({\mathbf{d}},{\mathbf{n}}) = 0 \hfill \\ \;\;\;\;\;\;{\text{Objective:}}\;\;{\text{minimizing}}\;\beta \hfill \\ {\text{Subject}}\;{\text{to:}}\;\;\beta = \beta_{T} \hfill \\ \end{gathered}$$

where **d** is the design parameter, *g*(**d**, **n**) is the limit state function. Based on Eq. (11), a series of reliability analyses need to be performed by varying **d** until the calculated *β* is equal to *β*_*T*_. It is noted that the requirement that ${\mathbf{x}} \in f$ in Eq. (9) is imposed as a constraint of *g*(**d**, **n**) = 0 in Eq. (11).

### Target reliability method

As the inverse of FORM, the procedure for target reliability method (TRM) has been demonstrated to be efficient and reliable (e.g.,^32–34^). In this study, the partial factors are also estimated using TRM. As shown in Fig. 3b, n* can be obtained by solving the following non-linear optimization problem:

12 $$\begin{gathered} {\text{Find:}}\;\;{\mathbf{n}} \hfill \\ {\text{Subject}}\;{\text{to:}}\;\;\beta = \beta_{T} \;{\text{and}}\;g({\mathbf{d}},{\mathbf{n}}) \ge 0 \hfill \\ {\text{Objective:}}\;\;{\text{minimizing}}\;g({\mathbf{d}},{\mathbf{n}}) \hfill \\ \end{gathered}$$

The ‘fmincon’ algorithm in Matlab is adopted herein to solve the aforementioned constrained nonlinear multivariable functions [i.e., Eqs. (11) and (12)]. Based on the solution results (i.e., n*) in Fig. 3, the partial factors corresponding to *β*_*T*_ can be computed as follows:

13 $$x_{i}^{ * } = F^{ - 1} \left[ {\Phi \left( {n_{i}^{ * } } \right)} \right]$$

14 $$\gamma_{{x_{i} }} = \frac{{x_{i}^{ * } }}{{\mu_{{x_{i} }} }}$$

where $x_{i}^{ * }$ is the equivalent design point of *x*_*i*_, $\gamma_{{x_{i} }}$ is the partial factor of *x*_*i*_, and $\mu_{{x_{i} }}$ is the mean value of *x*_*i*_.

### Ethical approval

The manuscript has been prepared by the contribution of all authors, it is the original authors work, it has not been published before, it has been solely submitted to this journal, and if accepted, it will not be submitted to any other journal in any language.

## Case study

To illustrate the proposed LRFD procedure for energy piles, an in-situ energy pile case^35,36^ is adopted in this study for demonstration. The related parameters for pile and soil are listed in Table 1. The pressuremeter test data are used herein to estimate the ultimate side resistance and ultimate base resistance of pile^37,38^. As the thickness of silts is small, this study mainly focuses on the variability of sands. In addition, the pressuremeter test data is normalized with depth and the detailed statistics are further characterized. Thus, the mechanical load (*P*_*M*_), pile-superstructure interaction stiffness (*K*_*h*_), normalized pressuremeter modulus of sands ($E_{M}^{n} = {{E_{M} } \mathord{\left/ {\vphantom {{E_{M} } z}} \right. \kern-\nulldelimiterspace} z}$), and normalized limit pressure of sands ($p_{l}^{n} = {{p_{l} } \mathord{\left/ {\vphantom {{p_{l} } z}} \right. \kern-\nulldelimiterspace} z}$) are selected as the uncertain parameters. Table 2 shows the statistics of those random variables. The annual temperature change of the example pile follows the cyclic heating and cooling mode in Fig. 2.

**Table 1:** Deterministic parameters for pile and soil.

Parameter	Notation		Value	
Pile	Diameter (m)	*D*	0.52	
	Length (m)	*L*	12	
	Young’s modulus (GPa)	*E*	20	
	Thermal expansion/contraction coefficient (°C^−1^)	*α*	1.2 × 10^–5^	
Soil	Ground water table (m)	*wt*	1.5	
	Layer	–	Silts	Sands
	Depth (m)	*z*	0‒2.7	> 2.7
	Unit weight (kN/m^3^)	*γ*	19.0	19.5
	Pressuremeter modulus (MPa)	*E*_*M*_	4	2.14*z*
	Limit pressure (MPa)	*p*_*l*_	0.5	0.26*z*

Data from Szymkiewicz et al.^36^ and Habert et al.^35^.

**Table 2:** Uncertain parameters used in the reliability analysis.

Parameter	Notation	Mean value	COV
Mechanical load (kN)	*P*_*M*_	1200	0.10^a^
Pile-superstructure interaction stiffness (GPa/m)	*K*_*h*_	2	0.10^a^
Normalized pressuremeter modulus of sands (MPa/m)	$E_{M}^{n}$	2.14^b^	0.33^b^
Normalized limit pressure of sands (MPa/m)	$p_{l}^{n}$	0.26^b^	0.15^b^

^a^Xiao et al.^21^; ^b^data characterized using field test results from Szymkiewicz et al.^36^ and Habert et al.^35^.

### Deterministic analysis

**Figure 4:**
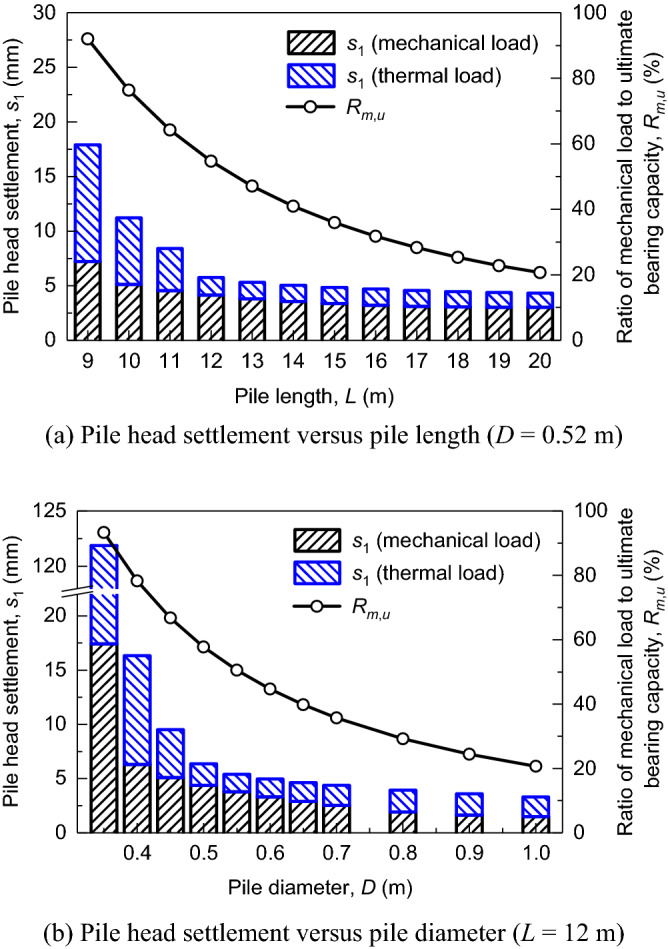
Performance of energy piles in different design scenarios.

**Figure 5:**
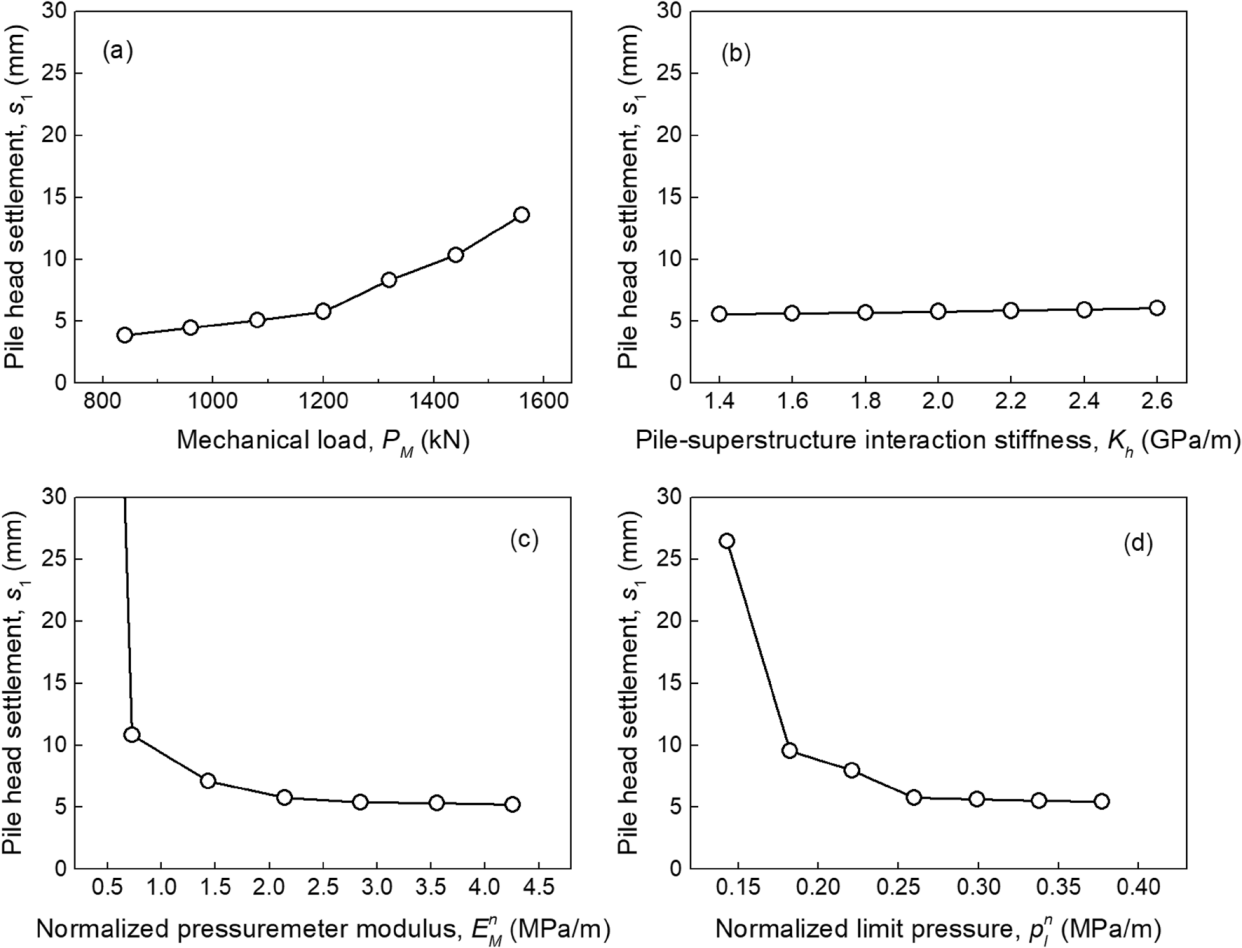
Effect of key parameters on the pile head settlement: (**a**) *P*_*M*_; (**b**) *K*_*h*_; (**c**) $E_{M}^{n}$; (**d**) $p_{l}^{n}$.

Based on the aforementioned load-transfer model for energy piles, a series of deterministic parametric analyses are conducted herein to investigate the effect of design parameters (${\mathbf{d}} = \left\{ {L,D} \right\}$) and critical parameters (${\mathbf{x}} = \left\{ {P_{M} ,K_{h} ,E_{M}^{n} ,p_{l}^{n} } \right\}$) on the long-term performance of energy piles. It is noted that the mean values of those random variables are used in the deterministic analysis. The number of pile element (*N*_0_) in the finite difference model is set to be 3000 through a convergence analysis to ensure both the computational accuracy and efficiency. In practice, the pile settlement can be controlled by changing its geometric dimensions. Figure 4 shows the pile head settlements (*s*_1_) at the end of 50 years along with the ratio of mechanical load to ultimate capacity (*R*_*m,u*_) in different design scenarios. For energy piles, *s*_1_ is induced by two types of loads, i.e., mechanical load and thermal load. It is worth noting that *s*_1_ induced by the mechanical load is consistent with that of a normal pile with no temperature variation. It is obvious that both *s*_1_ values decline with the increasing of *L* (or *D*) and finally stabilize. For example, the *s*_1_ values with pile length of 9 m (*R*_*m,u*_ = 92%), 12 m (*R*_*m,u*_ = 55%) and 20 m (*R*_*m,u*_ = 20%) are 18.0 mm, 5.7 mm and 4.3 mm, respectively. Similarly, when *L* = 12 m (Fig. 4b), the calculated *s*_1_ values vary from 6.4 mm to 3.3 mm when *D* increases form 0.50 m (*R*_*m,u*_ = 58%) to 1 m (*R*_*m,u*_ = 21%). In addition, the *s*_1_ values of a normal pile (i.e., black histogram in Fig. 4) are lower than those of an energy piles, especially when *R*_*m,u*_ exceeds 60%. Hence, it can be easily concluded that the cyclic thermal loading has a negligible impact on the pile head settlement. The accumulated pile settlement due to cyclic thermal loading can be well controlled by ensuring that the mechanical load is under 60% of the ultimate bearing capacity of energy piles. Furthermore, considering 95% interval bound (i.e., two standard bounds) of *P*_*M*_, *K*_*h*_, $E_{M}^{n}$, and $p_{l}^{n}$ (Table 2), the effect of those random parameters on *s*_1_ is investigated. As shown in Fig. 5, *s*_1_ values increase with the increasing of *P*_*M*_, whereas *s*_1_ appears to be little affected by *K*_*h*_. In addition, larger $E_{M}^{n}$ and $p_{l}^{n}$ lead to a smaller *s*_1_.

### Calibrating partial factors using first-order reliability method

**Figure 6:**
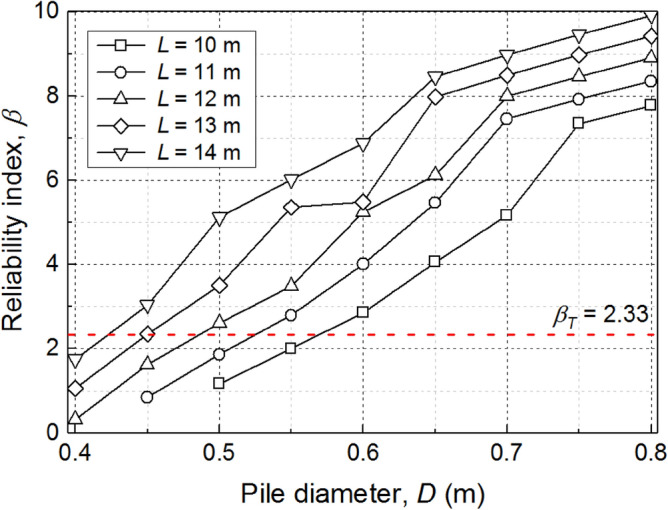
Reliability index estimated using first-order reliability method in different design scenarios.

Following the aforementioned FORM-based calibration procedure, a series of reliability analyses are firstly conducted. Considering the pile failure due to excessive settlement, the reliability index (*β*) at the end of 50 years is calculated for several combinations of *L* (10‒14 m) and *D* (0.4‒0.8 m). As shown in Fig. 6, *β* increases with the increasing of both *L* and *D*. For a given target reliability index (*β*_*T*_), the most probable values of the uncertain parameters (n*) are available from the results of FORM-based reliability analysis. When *β*_*T*_ = 2.33, the partial factors can be further calculated based on Eq. (14). As shown in Table 3, the calculated partial factors of mechanical load ($\gamma_{{P_{M} }}$), normalized pressuremeter modulus ($\gamma_{{E_{M}^{n} }}$) and normalized limit pressure ($\gamma_{{p_{l}^{n} }}$) vary in different design scenarios, whereas the change of the partial factor of the pile-superstructure interaction stiffness ($\gamma_{{K_{h} }}$) is negligible. The $\gamma_{{K_{h} }}$ value of 1.00 reveals that the uncertainty in *K*_*h*_ has little effect on the pile failure due to excessive settlement, which is similar to the observation in the previous deterministic analysis (Fig. 5b).

**Table 3:** Calibration results using first-order reliability method.

Design parameters		Results				
*L* (m)	*D* (m)	*β*	$\gamma_{{P_{M} }}$	$\gamma_{{K_{h} }}$	$\gamma_{{E_{M}^{n} }}$	$\gamma_{{p_{l}^{n} }}$
10	0.57	2.28	1.16	0.99	0.99	0.77
11	0.53	2.36	1.13	1.00	1.09	0.74
12	0.48	2.34	1.19	1.00	0.73	0.82
13	0.45	2.36	1.20	1.00	0.74	0.83
14	0.42	2.36	1.18	1.01	0.96	0.78

*L* is the pile length, *D* is the pile diameter, *β* is the reliability index, $\gamma_{{P_{M} }}$ is the partial factor of the mechanical load, $\gamma_{{K_{h} }}$ is the partial factor of the pile-superstructure interaction stiffness, $\gamma_{{E_{M}^{n} }}$ is the partial factor of the normalized pressuremeter modulus, $\gamma_{{p_{l}^{n} }}$ is the partial factor of normalized limit pressure.

### Calibrating partial factors using target reliability method

As noted previously, in the FORM-based calibration procedure, **d** need to be varied by trial-and-error until the calculated *β* is equal to *β*_*T*_. In contrast, the target reliability method (TRM) is a more efficient method to back-calculate the partial factors because there is no need of varying **d**. Hence, based on Eqs. (12)‒(14), the partial factors can be calculated by solving the constrained optimization problem [Eq. (12)]. It is noted that all the relevant parameters in the simulations are taken from Tables 1 and 2. The effects of pile-superstructure interaction stiffness, operational mode of energy piles, and design parameter on the calibrated partial factors (i.e., $\gamma_{{P_{M} }}$, $\gamma_{{K_{h} }}$, $\gamma_{{E_{M}^{n} }}$, and $\gamma_{{p_{l}^{n} }}$) are further investigated, respectively.

**Figure 7:**
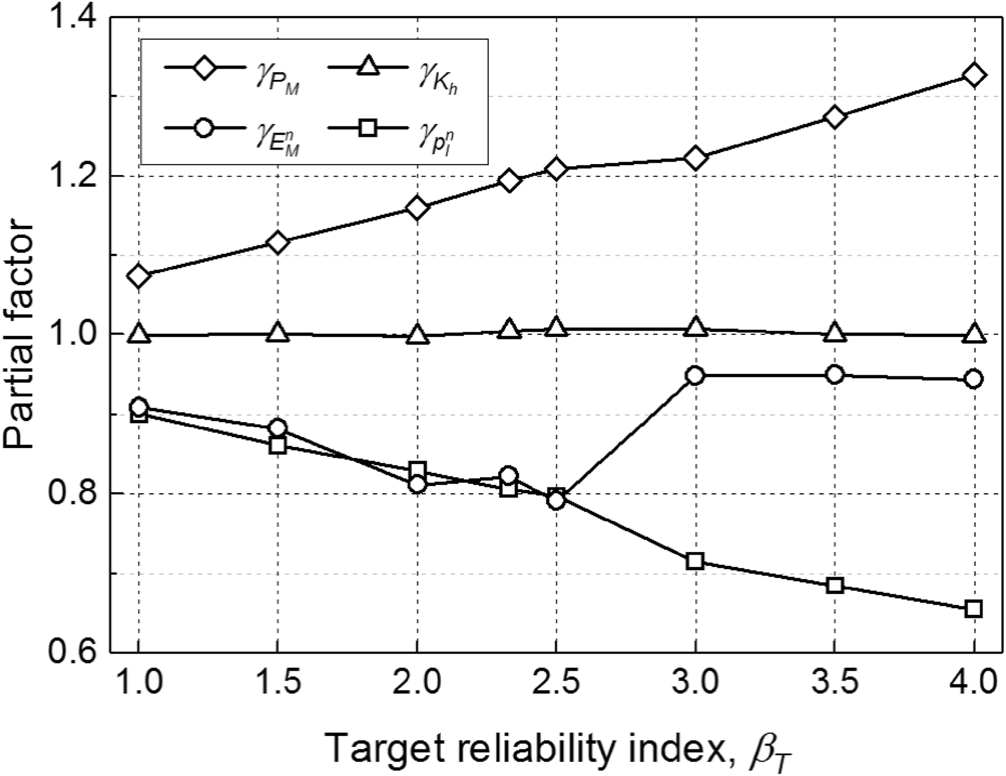
Calibrated partial factors for various target reliability indexes.

Following the cyclic heating and cooling load as shown in Fig. 2 (Δ*T* = 10 °C), a series of simulations based on the aforementioned TRM-based calibration procedure is performed. Figure 7 shows the calibrated partial factors for various target reliability index (*β*_*T*_). It is obvious that a larger *β*_*T*_ leads to a larger $\gamma_{{P_{M} }}$. This is mainly because a larger *β*_*T*_ means a higher safety requirement. Moreover, a trade-off relationship between $\gamma_{{E_{M}^{n} }}$ and $\gamma_{{p_{l}^{n} }}$ can be observed with the increasing of *β*_*T*_, while $\gamma_{{K_{h} }}$ remains approximately a fixed value of 1.00. As shown in Fig. 7, when *β*_*T*_ = 2.33, the values of $\gamma_{{P_{M} }}$, $\gamma_{{K_{h} }}$, $\gamma_{{E_{M}^{n} }}$, and $\gamma_{{p_{l}^{n} }}$ are 1.19, 1.00, 0.82, and 0.81, respectively. The calibration results using TRM are comparable with those using FORM (Table 3).

#### Effect of pile-superstructure interaction stiffness on the calibrated partial factors

**Figure 8:**
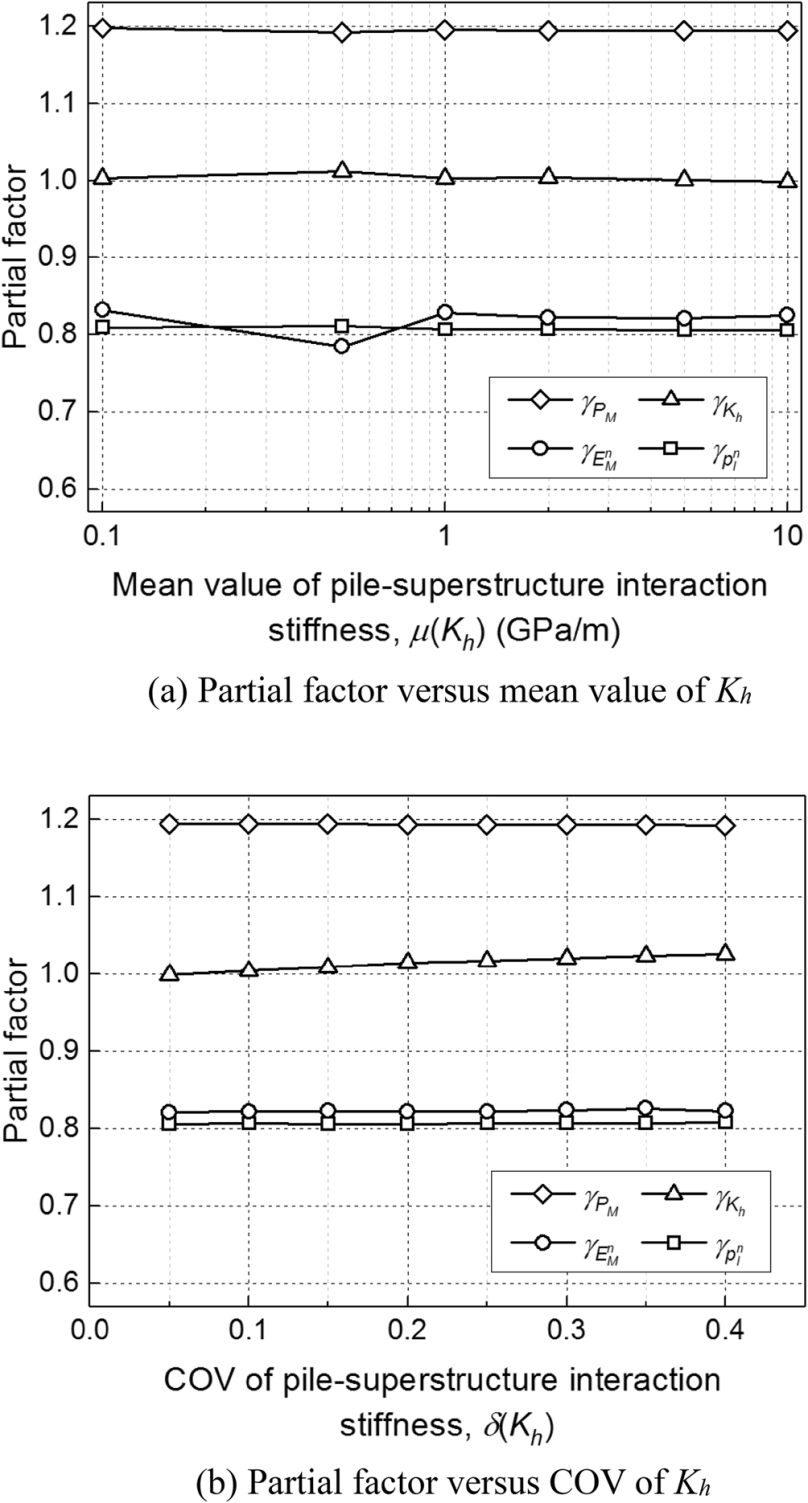
Effect of pile-superstructure interaction stiffness (*K*_*h*_) on calibrated partial factors.

To further explore the effect of pile-superstructure interaction stiffness (*K*_*h*_) on the calibrated partial factors, several other mean values [$\mu \left( {K_{h} } \right) = 0.1 - 10\;{\text{GPa/m}}$] and COVs ($\delta \left( {K_{h} } \right) = 0.05 - 0.40$) of *K*_*h*_ are considered. Following the cyclic heating and cooling load as shown in Fig. 2 (Δ*T* = 10 °C), a series of simulations based on the aforementioned TRM-based calibration procedure is performed (*β*_*T*_ = 2.33). As shown in Fig. 8, the change of partial factors with the increasing of $\mu \left( {K_{h} } \right)$ and $\delta \left( {K_{h} } \right)$ is negligible, which reveal that the partial factors of energy piles is less sensitive to the pile-superstructure interaction stiffness.

#### Effect of operational mode of energy piles on the calibrated partial factors

**Figure 9:**
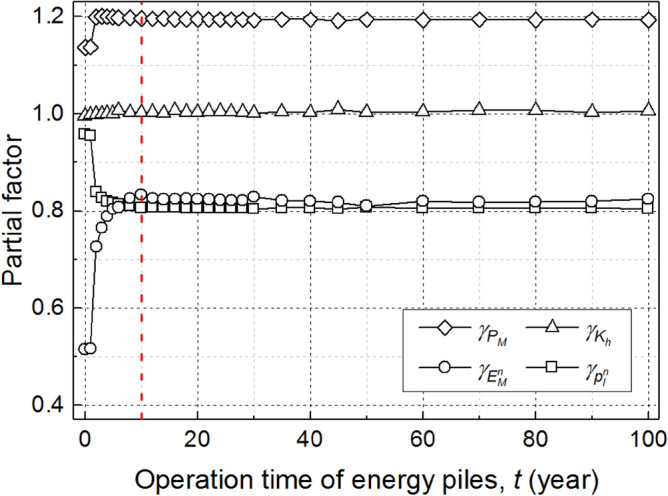
Effect of operation time of energy piles on calibrated partial factors.

In the service life of energy piles, the pile performance is directly affected by its operational mode [operation time (*t*) and temperature amplitude (Δ*T*)]. Similarly, taking *β*_*T*_ = 2.33 as an example and following the temperature cycle mode in Figs. 2, 9 shows the calibrated partial factors during 100-year operation time of the example energy pile. It is obvious that $\gamma_{{P_{M} }}$, $\gamma_{{E_{M}^{n} }}$, and $\gamma_{{p_{l}^{n} }}$ change gradually with *t*, while the change of $\gamma_{{K_{h} }}$ is negligible. In addition, when *t* > 10 year, the values of partial factors tend to stabilize, which reveal that the partial factors of energy piles is mainly affected by the thermal loading in the initial stage of service life.

**Figure 10:**
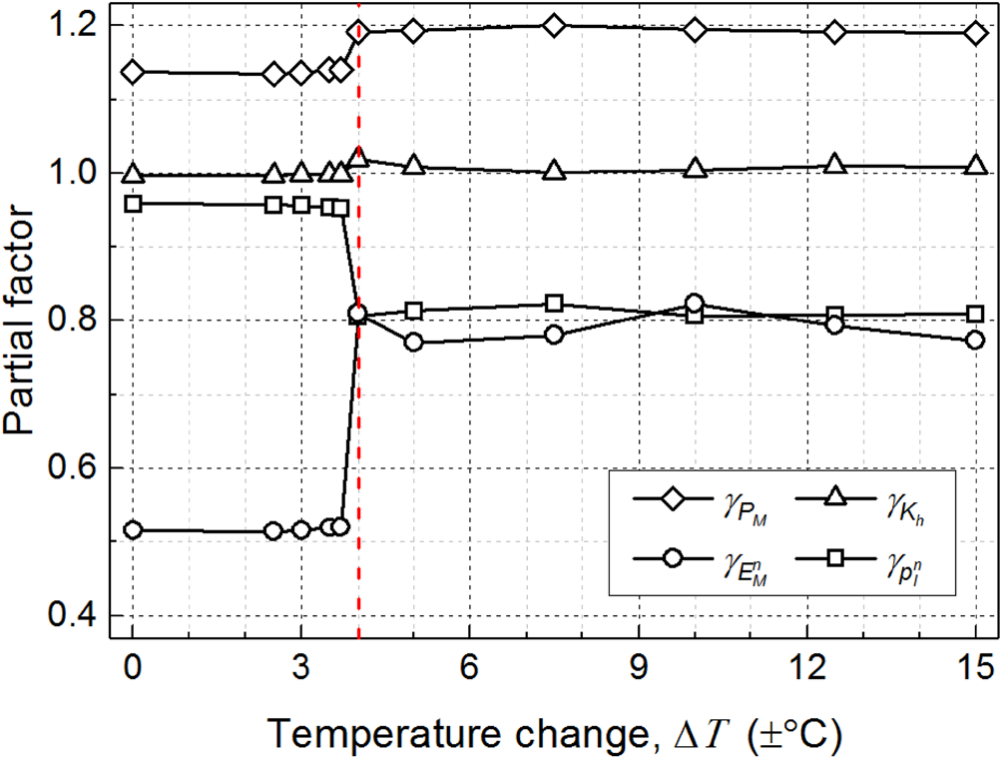
Effect of temperature change on calibrated partial factors.

To explore the effect of temperature change on the calibration results, several temperature amplitudes (Δ*T* ranges from 0 to ± 15 °C) are considered in the simulations. Similarly, based on Eqs. (12)‒(14), Fig. 10 shows the calibrated partial factors at the end of 50 years for various temperature changes (*β*_*T*_ = 2.33). It is obvious that the resulting partial factors are abruptly changed at the temperature change of ± 4 °C. The performance of the example energy pile is little affected by the thermal loading when Δ*T* is under ± 4 °C. As shown in Fig. 10, the values of $\gamma_{{P_{M} }}$, $\gamma_{{K_{h} }}$, $\gamma_{{E_{M}^{n} }}$, and $\gamma_{{p_{l}^{n} }}$ when Δ*T* is under ± 4 °C are similar to these when Δ*T* = 0 °C (i.e., traditional pile). In addition, when Δ*T* is higher than ± 4 °C, the change of partial factors with the increasing of Δ*T* is negligible. It can be easily concluded that the calibrated partial factors are robust against the temperature change when the temperature amplitude of energy piles is is large enough (Δ*T* >  ± 4 °C in this case study).

#### Calibrated partial factors in different design scenarios

The previous FORM-based calibration results (Table 3) revealed that the calibrated partial factors vary in different design scenarios. Hence, another series of pile lengths (*L* = 11‒20 m) and pile diameters (*D* = 0.50‒1.00 m) is considered in the simulations to explore the effect of design parameters on the calibrated partial factors. Taking *β*_*T*_ = 2.33 and Δ*T* =  ± 10 °C as an example, the calibrated partial factors at the end of 50 years can be obtained using TRM. As shown in Table 4, the changes of $\gamma_{{P_{M} }}$ and $\gamma_{{K_{h} }}$ with the increasing of *L* is negligible, while the values of $\gamma_{{E_{M}^{n} }}$ and $\gamma_{{p_{l}^{n} }}$ are abruptly changed when *L* > 13 m. Furthermore, it is obvious that the calibrated partial factors in each domain (i.e., *L* ≤ 13 m and *L* > 13 m) are similar to each other. Furthermore, the calibrated factors for various *D* are listed in Table 5. Similar to the observations in Table 4, the values of $\gamma_{{P_{M} }}$, $\gamma_{{K_{h} }}$, $\gamma_{{E_{M}^{n} }}$, and $\gamma_{{p_{l}^{n} }}$ can be categorized into two parts: *D* ≤ 0.60 m and *D* > 0.60 m. It can be easily concluded that the design parameters have a significant impact in the LRFD of energy piles. In practice, the partial factors need to be calibrated in different design scenarios and the worst values of each partial factor can be selected as the final results. For example, based on the results from Tables 4 and 5, the final values of $\gamma_{{P_{M} }}$, $\gamma_{{K_{h} }}$, $\gamma_{{E_{M}^{n} }}$, and $\gamma_{{p_{l}^{n} }}$ for *β*_*T*_ = 2.33 are taken as 1.19, 1.03, 0.50, and 0.79 in this study, respectively.

**Table 4:** Effect of pile length (*L*) on the partial factors calibrated using target reliability method (*D* = 0.52 m).

*L* (m)	*g*(**d**, **u**)	$\gamma_{{P_{M} }}$	$\gamma_{{K_{h} }}$	$\gamma_{{E_{M}^{n} }}$	$\gamma_{{p_{l}^{n} }}$
12	10.26	1.19	1.00	0.82	0.81
11	1.41	1.19	1.00	0.73	0.82
13	15.13	1.19	1.01	0.89	0.80
14	17.81	1.15	1.00	0.54	0.94
15	18.44	1.15	1.00	0.53	0.95
16	18.88	1.15	1.00	0.53	0.95
17	19.22	1.16	1.00	0.54	0.95
18	19.49	1.17	1.00	0.55	0.95
19	19.70	1.18	1.00	0.57	0.95
20	19.88	1.18	1.01	0.58	0.94

**Table 5:** Effect of pile diameter (*D*) on the partial factors calibrated using target reliability method (*L* = 12 m).

*D* (m)	*g*(**d**, **u**)	$\gamma_{{P_{M} }}$	$\gamma_{{K_{h} }}$	$\gamma_{{E_{M}^{n} }}$	$\gamma_{{p_{l}^{n} }}$
0.52	10.26	1.19	1.00	0.82	0.81
0.50	5.40	1.19	1.01	0.77	0.82
0.55	14.95	1.19	1.03	0.80	0.81
0.60	20.13	1.18	1.03	0.89	0.79
0.65	24.70	1.12	1.00	0.50	0.95
0.70	27.66	1.13	1.01	0.51	0.95
0.75	30.58	1.13	1.01	0.51	0.94
0.80	33.45	1.13	1.01	0.51	0.94
0.85	36.30	1.14	1.01	0.52	0.93
0.90	39.12	1.14	1.01	0.53	0.93
0.95	41.92	1.14	1.01	0.53	0.92
1.00	44.70	1.15	1.01	0.54	0.92

## Conclusions

This paper presents a reliability-based load and resistance factor design (LRFD) approach for energy piles considering the long-term performance degradation. Based on the load-transfer model, the serviceability limit state (settlement) of energy piles is investigated. The developed LRFD procedures are demonstrated to be effective through a case study. Based on the work presented, the following conclusions are drawn:

1. The LRFD model based on target reliability method (TRM) and first-order reliability method (FORM) are implemented into two different constrained nonlinear optimization problems, respectively. The partial factors calibrated using TRM are comparable with those using FORM, whereas TRM is an easier-to-use tool because there is no need of varying design parameters.
2. The operational mode of energy piles has a significant impact on the LRFD results. In the service life of energy piles, the calibrated partial factors change obviously in the initial stage and finally stabilize with time. Furthermore, the pile performance is little affected with the thermal loading when the temperature change is low (Δ*T* ≤  ± 4 °C), while the calibrated partial factors appear to be less sensitive to temperature amplitudes if Δ*T* is high enough (Δ*T* >  ± 4 °C).
3. The partial factors of energy piles are robust against the pile-superstructure interaction stiffness in this case study. In addition, the calibrated partial factors vary in different design scenarios and the worst values of each partial factor are recommended as the final results in practice.

## Data Availability

The datasets used and/or analysed during the current study available from the corresponding author on reasonable request.
